# Smoking, Suicidality and Psychosis: A Systematic Meta-Analysis

**DOI:** 10.1371/journal.pone.0138147

**Published:** 2015-09-15

**Authors:** Anoop Sankaranarayanan, Serafino Mancuso, Helen Wilding, Suhaila Ghuloum, David Castle

**Affiliations:** 1 Department of Geriatrics, Hamad Medical Corporation, Doha Qatar; 2 Department of Psychiatry, Weil Cornell Medical College, Doha Qatar; 3 St Vincent’s Hospital, Melbourne, Australia; 4 Department of Psychiatry, The University of Melbourne, Melbourne, Australia; 5 Department of Psychiatry, Hamad Medical Corporation, Doha Qatar; 6 Department of Psychiatry, Australian Catholic University, Melbourne, Australia; University of Vienna, School of Psychology, AUSTRIA

## Abstract

The aim of this study is to systematically review the literature that explored the association between smoking and suicidal risk among those with serious mental illness and to estimate the risk of suicidal behaviors attributable to smoking among this patient group. Multiple databases (CINAHL, PsycINFO, EMBASE, Informit Health Collection and the Cochrane Library databases) were searched from 1 January 1975 through 15 January 2014, along with references from relevant articles for observational studies that ascertained the association between smoking and suicidal behaviors among patients with psychotic disorders conducted in adult patients. Thirteen studies involving 6813 patients with severe mental illness were included. We found that smoking was significantly associated with suicidality in psychosis with an Odds Ratio of 2.12 (95% CI 1.67–2.7). Smoking is associated with suicidal risk amongst individuals with a severe mental illness; however, it is still unclear whether this represents a true risk factor or a confounder or a mediator via mechanisms, hitherto unknown, needs to be studied further.

## Introduction

Although there are differences in smoking rates and anti-smoking campaigns between different countries, it would be safe to surmise that smoking awareness is overall higher than it has been in the past; despite this, people continue to smoke. People with mental illness are more likely to smoke than the general population. According to large epidemiological studies the prevalence of smoking is about 2–3 fold higher in those with a mental illness [1]. The risk of smoking is particularly high in those with severe mental illnesses such as schizophrenia, bipolar disorders and depression [2–8]. Individuals with these disorders also smoke, on average, more cigarettes than the general population [9, 10] and are less likely to quit [11]. An elevated risk for smoking has also been reported in those with alcohol or substance use and anxiety disorders [12, 13].

Research undertaken to understand why people with psychosis smoke has focused mainly on the “self-medication hypothesis”. According to this hypothesis smoking alleviates negative mood states, reduces positive symptoms, enhances cognitive functioning and reduces medication-induced side effects [14–17]. Others contend that this association is best explained by a “shared diathesis” between tobacco and mental illness, and provide evidence for how smoking predates psychosis [18].

A number of studies have focused on the association between smoking and increased suicidal risk [19–21]. Some such studies have specifically addressed this issue in mentally ill cohorts [22–24]. Interestingly, while studies in general population samples have found an association between smoking and an increased risk of suicide, as evidenced by a recent meta-analysis [25], studies done in mentally ill have yielded conflicting results. For example, while Tanskanen et al [22] and Kessler et al [26] have shown a positive association between smoking and suicidality among patients with mental illness, our study [27] failed to demonstrate an increased suicidal risk in smokers with a psychotic disorder, particularly after adjusting for confounding factors such as depression.

To interrogate these inconsistencies, we conducted a systematic-review and meta-analysis of observational studies to determine the effect of smoking on risk of suicide or suicidal behaviour in people with psychotic disorders. Our apriori hypothesis was that smoking is associated with an increased risk (current or lifetime) of suicidality (suicide ideation, suicide attempts, or completed suicides) in people with a psychotic disorder.

## Materials and Methods

We have followed the Preferred Reporting Items for Systematic Reviews and Meta-Analysis (PRISMA) statement [28] in reporting this systematic review (detailed in S1 PRISMA Checklist). Studies were identified by searching six electronic databases and checking reference lists of relevant articles. Results were limited to articles published in the English language between 1 January 1975 and 15 January 2014. Database searches were carried out by an experienced medical research librarian (HW) with an initial search strategy applied to Medline (EBSCOhost) and adapted for CINAHL (EBSCOhost), PsycINFO (EBSCOhost), EMBASE (Embase.com), Informit Health Collection and the Cochrane Library. All searches were last updated on 15 January 2014. All potentially eligible studies were considered for review.

The search strategy for Medline (EBSCOhost) used a combination of MeSH Terms and title keywords as directed by our research question. Accordlngly, we were interested in studying the association between smoking [(MH "Smoking") OR (MH "Tobacco") OR (MH "Tobacco Use Cessation") OR (MH "Smoking Cessation") OR (MH "Tobacco Use Disorder") OR (MH "Nicotine") OR TI smoking OR TI cigarette* OR TI nicotine OR TI tobacco] and all suicidal behaviors [(MH "Suicide") OR (MH "Suicidal Ideation") OR (MH "Suicide, Attempted") OR (MH "Self-Injurious Behaviour") OR (MH "Self Mutilation") OR TI suicid* OR TI self-harm* OR TI DSH OR TI self-mutilat* OR TI self-injur*] and patients with severe mental illness [(MH "Mental Disorders") OR (MH "Anxiety Disorders+") OR (MH "Eating Disorders+") OR (MH "Mood Disorders+") OR (MH "Schizophrenia and Disorders with Psychotic Features+") OR (MH "Personality Disorders+") OR TI mental OR TI psychiatric OR TI psychosis OR TI psychotic OR TI schiz* OR TI mood disorder* OR TI affective disorder* OR TI bipolar OR TI depression OR TI depressive disorder* OR TI personality disorder* OR TI eating disorder* OR TI anorexia OR TI bulimia]

We used a broader search strategy that included mental illnesses other than psychosis as some studies include multiple diagnoses and we were keen to screen their appropriateness for the study. Similarly we wanted to include all forms of suicidal behaviors that had previously been associated with smoking. Previous systematic reviews on suicidal behaviors have used a similar approach [29, 30].

Other searches followed a similar format with adaptations appropriate to the individual database; thesaurus and subject headings used i.e. CINAHL Headings and EMTREE Terms. The additional search strategies are set out in the Appendix.

### Study Selection

Studies were considered eligible and included if they fulfilled the following criteria:

1. Observational studies
2. Included adults (above the age of 18 years),
3. Performed in patients with a psychotic disorder; for the purpose of this review, we included the following diagnostic categories: schizophrenia, schizoaffective disorder, first episode psychosis, delusional disorder, bipolar disorder and psychotic depression.
4. Studies that specifically looked for or reported an association between cigarette smoking and suicidality; we included studies that reported suicidal risk, suicide attempt, suicidal ideation and/or completed suicide.

### Data Extraction and Quality Assessment

AS and SG independently reviewed study titles and abstracts for potential inclusion in the systematic review. Studies that satisfied the inclusion criteria were retrieved for full-text evaluation. Studies selected for detailed analysis by these two authors had an agreement value (Κ) of .88; a third investigator (DC) resolved disagreements.

We developed a 19-item data-extraction sheet, based on the STROBE statement [31], that would yield a maximum of 23 points based on the type of study, clarity of aims, objectives and hypothesis, internal validity, external validity and statistical validity. This is detailed in S2 Table. While this would naturally award higher points for cohort studies, we also factored in negative markings where cohort studies did not discuss drop-out rates or fixed time periods of assessments. Further, we adjusted the final score by subtracting one point when studies did not score across all categories. Where two studies had the same adjusted scores, the final rating also took into consideration the type of study and the sample size. We also extracted quantitative data including characteristics of the sample, number of events and unadjusted and adjusted risk estimates. Where the study did not provide specific information, publication’s authors were contacted by e-mail for extra information.

### Meta-analysis

The included studies employed a range of outcome measures to examine the association between smoking and suicidality. Some reported the exact number of suicidal behaviours (i.e., suicide ideation, suicide attempts, or completed suicides) in people with a psychotic disorder, while others reported odds ratios. We used odds ratios with 95% confidence intervals as the main outcome measure. To calculate the pooled odds ratio, we used R, version 3.0.2 (R foundation for Statistical Computing, Vienna, Austria) with the ‘metafor’ package [32]. Due to differences in study design, sample characteristics, and outcome measures, we expected considerable heterogeneity amongst the studies. Therefore, we calculated the pooled odds ratio using a random-effects model with the DerSimionian-Laird estimator [33].

To examine heterogeneity, we calculated the I^2^ statistic and its 95% confidence intervals. A value of 0% indicated no observed heterogeneity, and larger values show increasing heterogeneity, with 25% considered low, 50% moderate, and 75% high heterogeneity [34]. We tested for heterogeneity using the Q statistic, with p<0.10 indicating significant heterogeneity.

Subgroup analyses were conducted using the mixed-effects models function of the ‘metafor’ package [32]. For this method, studies within groups are pooled with the random-effects model, while tests for significant differences between subgroups are conducted with the fixed-effects model.

Publication bias was assessed using funnel plots and the trim-and-fill procedure [34], which yields an estimate of the effect size after publication bias has been taken into account. We also conducted Egger’s regression test [35] to quantify the bias captured by the funnel plot and tested whether it was significant. All results were considered significant at p<0.05.

## Results

**Fig 1:**
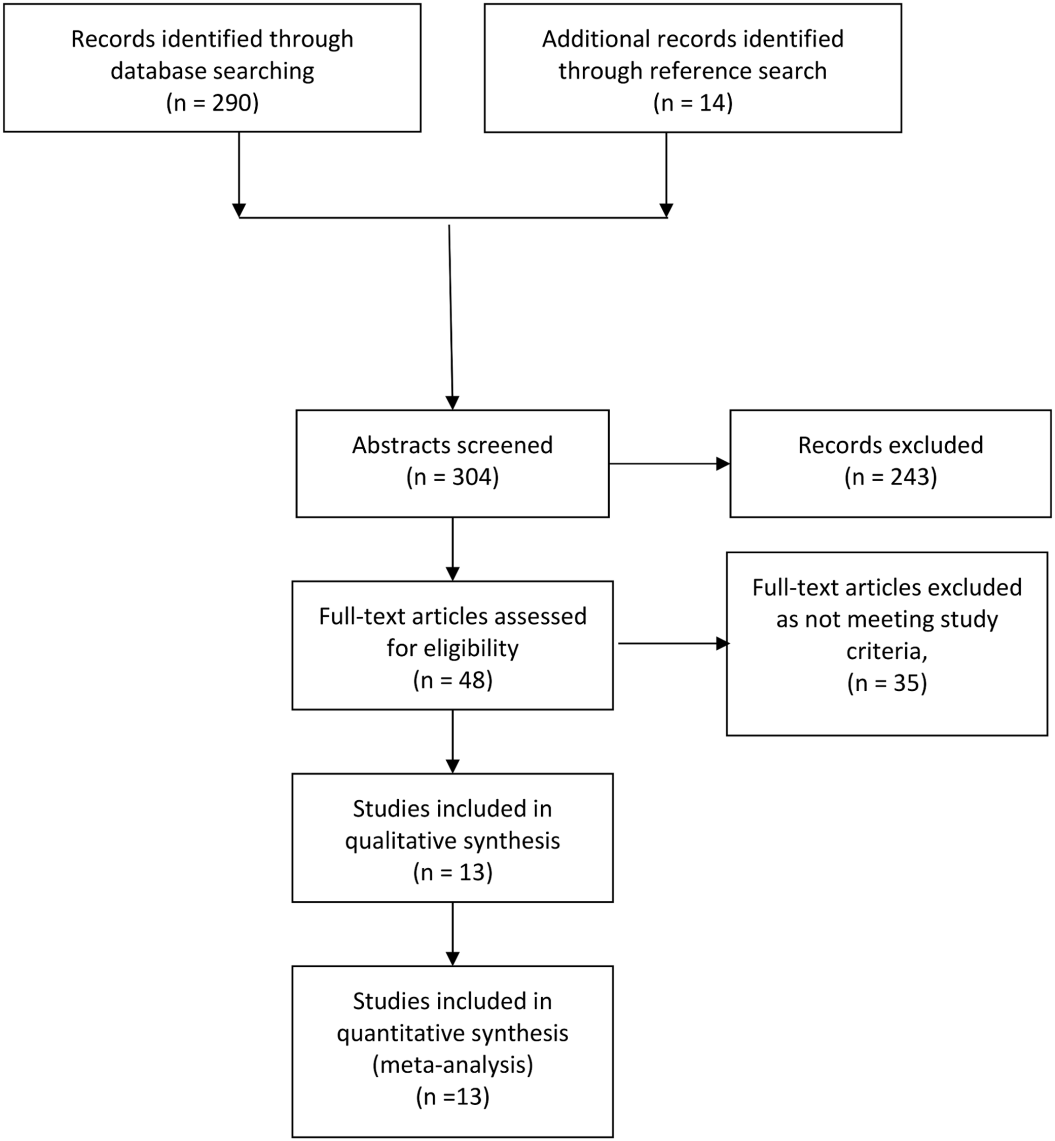
Flow-diagram for screening of articles.

Fig 1 details the search-strategy flow-chart. We identified a total of 13 studies [6; 23; 27; 36–45] meeting our predefined criteria. Seven were cross-sectional, two case-control and four were cohort studies. While two studies [27; 37] employed a broad definition for psychotic illnesses and included patients with schizophrenia, schizoaffective disorders, bipolar disorders and psychotic depression, other studies focused solely on either schizophrenia (and/or schizoaffective disorder) or bipolar disorder. Only one study [38] ascertained completed suicides as the outcome of interest. Other studies focused on suicide attempts and/or suicidal intent. The studies are summarised in S1 Table.

S3 Table summarizes the qualitative assessments for the studies. Five studies [6; 23; 27; 40; 42] reported a clear hypothesis. The study of Ostacher et al [39] received the highest rating. Five studies [6; 38; 39; 42; 45] rated poorly on internal validity rating, thereby questioning their final conclusions. Three studies [23; 38; 42] did not describe the final adjusted odds ratio.

We aimed to study whether smoking is associated with an increased risk (current or lifetime) of suicidality (suicide ideation, suicide attempts, or completed suicides) in people with a psychotic disorder. Six studies [6; 36. 39; 43; 37; 45] reported suicide attempt, while the rest studied both suicidal ideation and suicidal attempts. Of the studies that reported rates of suicidal attempts, all except one [36] reported lifetime rates. Altamura et al (2003) reported current rates.

### Meta-analysis

**Fig 2:**
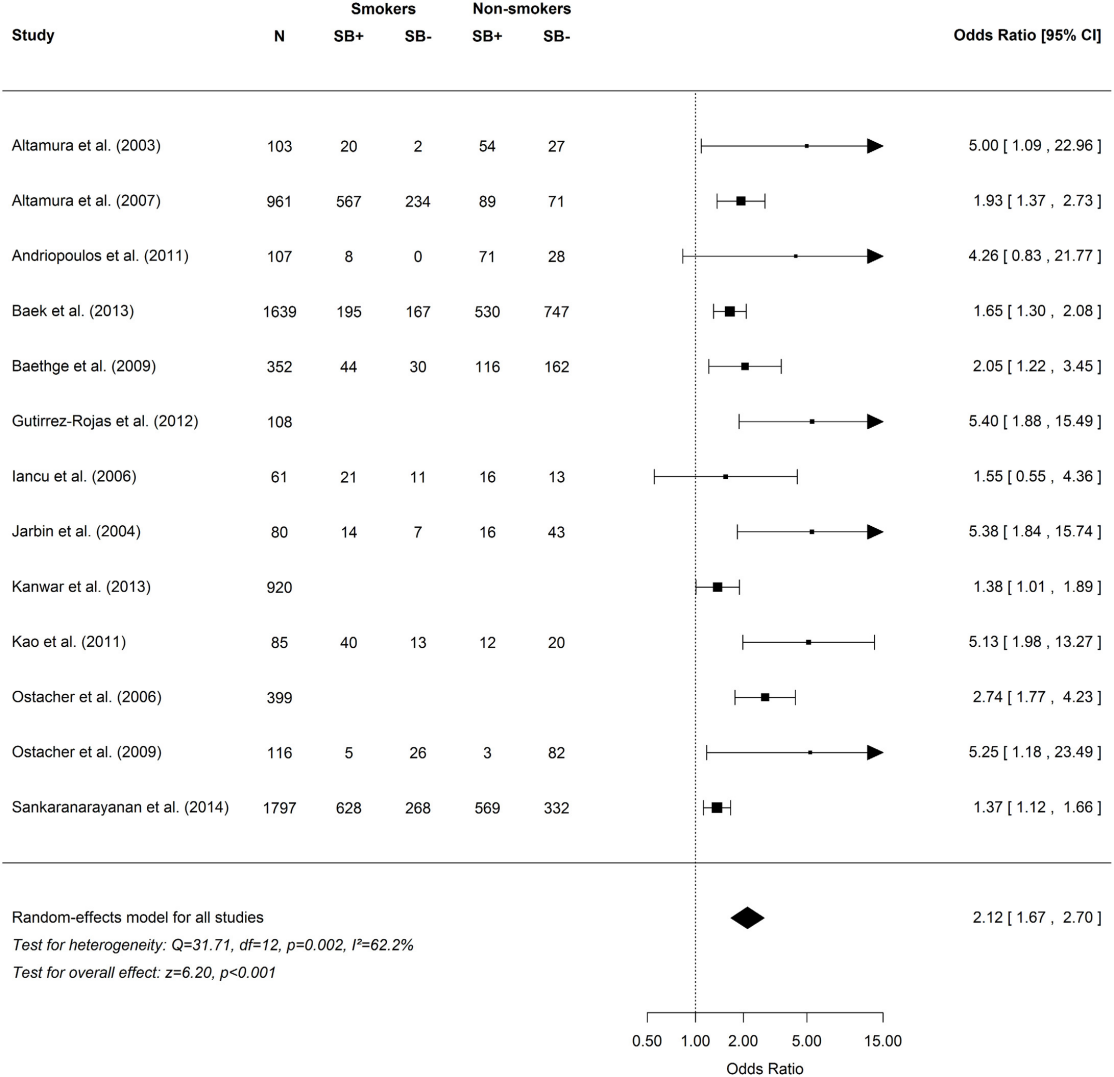
Frequency table and forest plot for suicidal behaviors in psychosis meta-analysis.

As shown in Fig 2, the overall odds ratio for the association between cigarette smoking and suicidality in psychosis was 2.12 (95% CI = 1.67–2.70, p<0.001). Heterogeneity was moderate and significant (I^2^ = 62.2%, 95% CI = 28.5–93.0, p = 0.002). Exclusion of three potential outliers (27; 37; 44) significantly reduced heterogeneity (I^2^ = 25.0%, 95% CI = 0.0–81.0, p = 0.213) and had a small effect on the overall odds ratio (2.74, 95% CI = 2.07–3.62, p<0.001).

**Fig 3:**
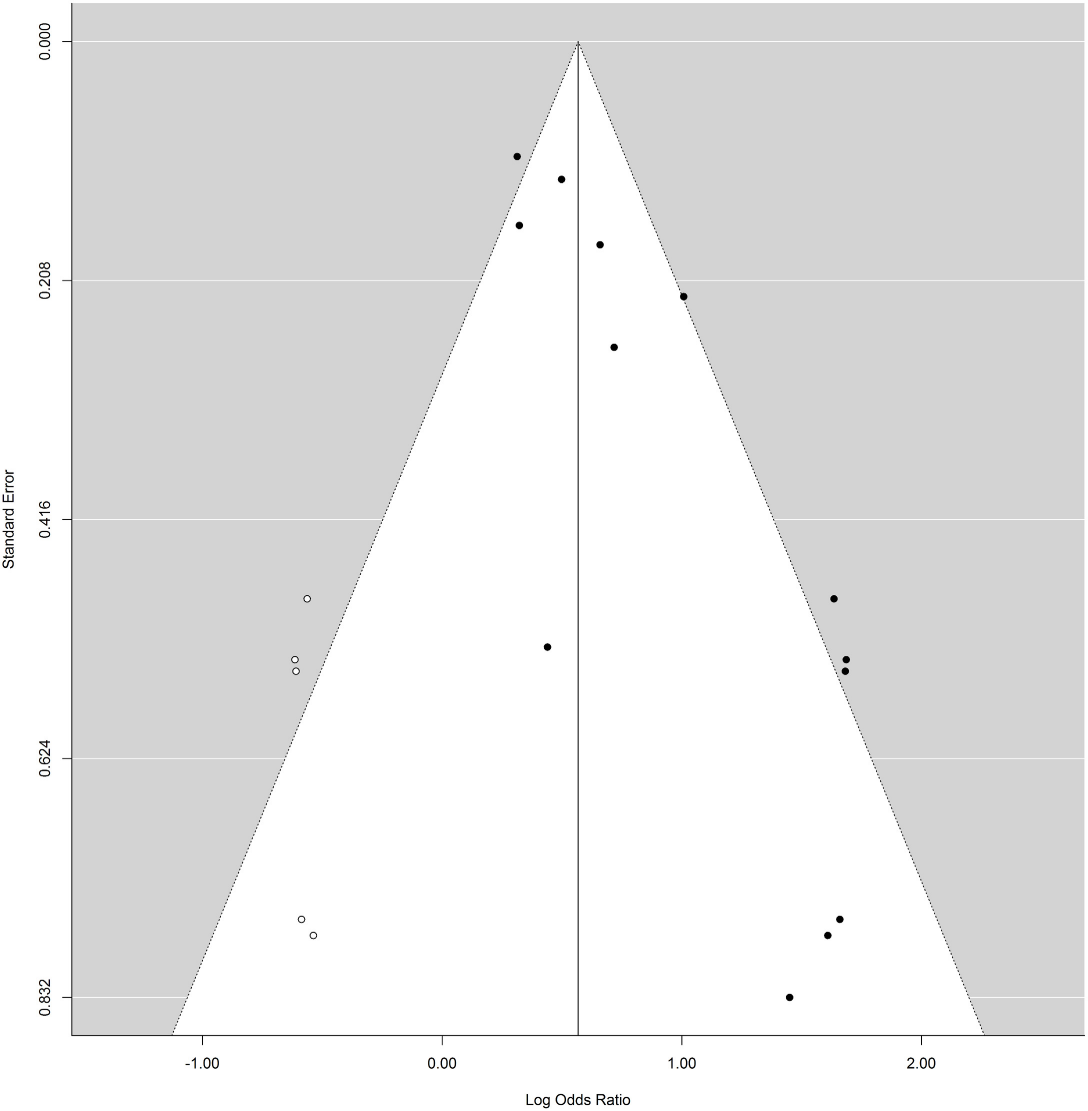
Trim-and-fill plot.

After adjustment for publication bias with the trim-and-fill procedure, the overall odds ratio was reduced to 1.76 (95%CI = 1.37–2.26, p<0.001; the number of filled studies was 5). Egger’s regression test for funnel plot asymmetry also indicated significant publication bias (z = 4.65, p<0.001). Given this small reduction in the overall odds ratio, which remained significant, the impact of publication bias is likely modest [46]. A funnel plot with the imputed studies is presented in Fig 3.

#### Subgroup analyses

The subgroup analyses are presented in Table 1, which shows that the odds ratio did not differ between suicidal behavior type (suicide attempt and. suicide attempt/suicidal ideation) (p = 0.421). The odds ratio did not differ in relation to diagnostic group (schizophrenia/schizoaffective disorder, bipolar disorder, and broad psychosis) (p = 0.851) or study design (case-control, cohort, and cross-sectional) (p = 0.243).

**Table 1:** Meta-analyses results for random-effects and mixed-effects models.

	Effect Size		Heterogeneity		p
	OR	(95% CI)	I^2^	(95% CI)	
Random-effect models					
Unadjusted	2.12	(1.67–2.70)***	62.2%	(28.5–93.0)**	
Outliers removed	2.74	(2.07–3.62)***	25.0%	(0.0–81.1)	
Adjusted for publication bias	1.76	(1.37–2.26)***	25.0%	(0.0–81.0)***	
Suicidal Behaviours					0.421
Attempts	2.63	(1.66–4.16)***	67.7%	(12.1–95.3)***	
Attempts/Ideation	1.84	(1.40–2.42)***	54.7%	(0.0–97.1) **	
Sample					0.851
Schizophrenia/Schizoaffective Disorder	2.12	(1.40–3.19)***	52.1%	(0.0–95.2)^+^	
Bipolar Disorder	2.12	(1.32–3.42)**	59.7%	(0.0–99.6)^+^	
Broad Psychosis	2.44	(0.65–9.18)	83.4%	(16.8–99.9)*	
Design					0.243
Case-control	2.09	(0.85–5.18)	5.1%^a^		
Cohort	2.76	(2.03–3.76)***	0.8%	(0.0–90.1)	
Cross-sectional	1.77	(1.37–2.29)***	67.4%	(31.0–98.8)**	

^a^ Unable to calculate confidence interval for I^2^ (*k* = 2).

^+^ p<0.10

* p<0.05

** p<0.01

*** p<0.001

## Discussion

While smoking has been associated with suicidal behaviours in the general population, studies limited to those with a mental illness have shown conflicting results. We undertook a systematic review and meta-analysis to study the strength of association reported in observational studies conducted in people with a psychotic illness. We found that smoking was significantly associated with suicidal behaviours among people with severe mental illness. Accordingly, the pooled odds ratio for the association was 2.12. We did not find differences in the odds ratio across study design (i.e., case-control, cohort, and cross-sectional) or diagnosis (i.e., schizophrenia/schizoaffective disorder, bipolar disorder, and broadly defined psychosis). This strongly implies that there is a significant association between smoking and suicidal behaviors in people with severe mental illness thereby proving our hypothesis right.

Smoking is highly prevalent among people with severe mental illness; for example, a meta-analysis based on 9 studies from 6 countries [11] demonstrated that patients with schizophrenia have a higher prevalence of ever smoking compared to the general population. Smoking has also been shown to be associated with suicidal risk in a previous meta-analysis [25]. It is therefore conceivable that smoking is associated with suicidal behaviours among patients with severe mental illness. Such an association was first described nearly four decades ago [19] although it was downplayed as an “artefact” [47].

From a clinical point, what is more important though is to explain this association; for example, is smoking a predictor or an independent risk factor or a mediator or in fact a confounder. Hughes [48] reviewed the association between smoking and suicide and proposed three potential explanatory hypotheses: (i) smoking is a non-causal marker; (ii) smoking is a psychological or physical toxin; and (iii) smokers are self-medicating a suicide risk.

If indeed smoking was “suicidogenic”, we need to understand the possible mechanisms. One approach therefore would be to tease out the effects of smoking that might contribute to suicidal behaviors in those with mental illness. Previous research indicates that smoking might increase the risk of mental illness [49–53] possibly through its action on brain nerurotransmitters such as serotonin [54] or Monoamine Oxidase [55]. It is also possible that smoking and mental illness shares common vulnerability factors [56–58].

Such individuals with a predisposition might begin to smoke as a form of coping strategy [59]; for example, patients with severe mental illnesses have significant problem solving difficulties, particularly in the social context [60–66]. It is likely that smoking leads to some initial gains, such as improved attention, behavioural arousal and enhanced problem-solving [67–68]; however chronic smoking can lead to neurocognitive deficits such as impairments in cognitive flexibility. Cognitive inflexibility is defined as the inability to change decision-making in response to feedback from the environment [69]. Such individuals have difficulty in finding effective solutions to problems during times of stress, which leads to hopelessness and suicidal ideation and/or tend to brood or in other words, have difficulty disengaging from negative ruminations [70]. The diathesis–stress–hopelessness model of suicidality [71–72] identifies hopelessness as a key mechanism through which cognitive inflexibility results in suicidal ideation [73–74].

From a biological point of view, chronic smoking is associated with neurobiological changes such as orbito-frontal cortical (OFC) thinning [75–76] and/or biochemical changes such as lower serotonin [77–78]. These changes are significant because lesion studies have demonstrated that emotion recognition and other social cognitive abilities depend critically on the orbitofrontal cortex [79–81] while suicidal behaviours, aggression, impulsivity and smoking have all been linked to a reduced central serotonin or “low serotonin syndrome” [82]. These changes affect the individual’s decision making capacity, impulse control, judgment, planning and reasoning skills, and hence serve to increase the risk of suicidal behaviours [54].

In summary, it is plausible therefore that patients with severe mental illness (or a vulnerability thereto) starts smoking either because they share common heritable factors or as a coping strategy to manage certain distress (e.g. negative affect). However chronic smoking is associated with biological and neurocognitive changes that in turn predispose the individual to contemplate suicide and/or engage in such behaviors because of increased impulsivity and/or poor problem solving abilities.

### Study Limitations

The results of our study must be interpreted within the context of some limitations. Firstly, we only chose literature published in English and this is likely to cause selection bias. Secondly, there was significant heterogeneity across studies. However, removal of potential outliers reduced heterogeneity to non-significance but had only a small effect on the overall odds ratio. In a similar vein, although there was evidence of publication bias, smoking was still significantly associated with suicidality in patients with severe mental illness after adjusting for this bias.

Our finding of a significant association between smoking and suicidality in those with severe mental illness is not in keeping with findings from previous systematic reviews on suicidal risk factors in severe mental illness [83–88]. Interestingly, while Hor and Taylor [89] reported a weak association between smoking and suicide, they did not elaborate on this. It is likely that the earlier reviews included studies that adjusted for smoking and therefore did not find a significant association. Leistikow [90] had warned against “over-adjusting” as smoking may independently be *associated* with and contribute to other suicide risk factors that the authors adjust for, including adverse life situations and stress.

Finally, although we were keen on studying all suicidal behaviors including completed suicide, we could identify only one study [44] that described completed suicide as an outcome of interest. Thus we did not have the capacity to study completed suicide and have only reported on suicidal behaviors. While suicidal behaviours are doubtlessly an important “predictor” of future suicide, the predictive capabilities are limited as suicide is rare. For example, the association between suicidal ideation and completed suicide is weak [91].

## Conclusions

Smoking is moderately associated [92] with suicidal behaviors in patients with psychotic disorders as is evidenced by results of our meta-analysis. What is less well known is how smoking is associated with suicidality in this patient group. There is however potential to test the effectiveness of smoking intervention in reducing suicidality in this patient group. Future research should therefore focus on the impact of smoking cessation on suicidal behaviours in those with psychotic disorders. Further, it would also be useful to test some of these theories in clinical research to build on the evidence base. Examples include studying neurocognitive functioning in smokers who attempt suicide and in particular looking for any correlation to neuroimaging findings. This can, however, be difficult to undertake considering there are multiple potential confounders. Ideally one would need a long-term prospective study that describes all variables of interest and can adjust for association between and interaction of changes in smoking status, mental status, and suicidality over time.

## Supporting Information

S1 PRISMA Checklist PRISMA checklist. (DOC)

S1 Table Summary of Studies included in the Meta-Analysis. (DOC)

S2 Table Guide to Quality Rating. (DOC)

S3 Table Summary of Quality Ratings. (DOC)
