# Correction: Smoking, Suicidality and Psychosis: A Systematic Meta-Analysis

**DOI:** 10.1371/journal.pone.0141024

**Published:** 2015-10-15

**Authors:** Anoop Sankaranarayanan, Serafino Mancuso, Helen Wilding, Suhaila Ghuloum, David Castle

Fig 1 is incorrect. Please see the corrected Fig 1 here.

**Fig 1:**
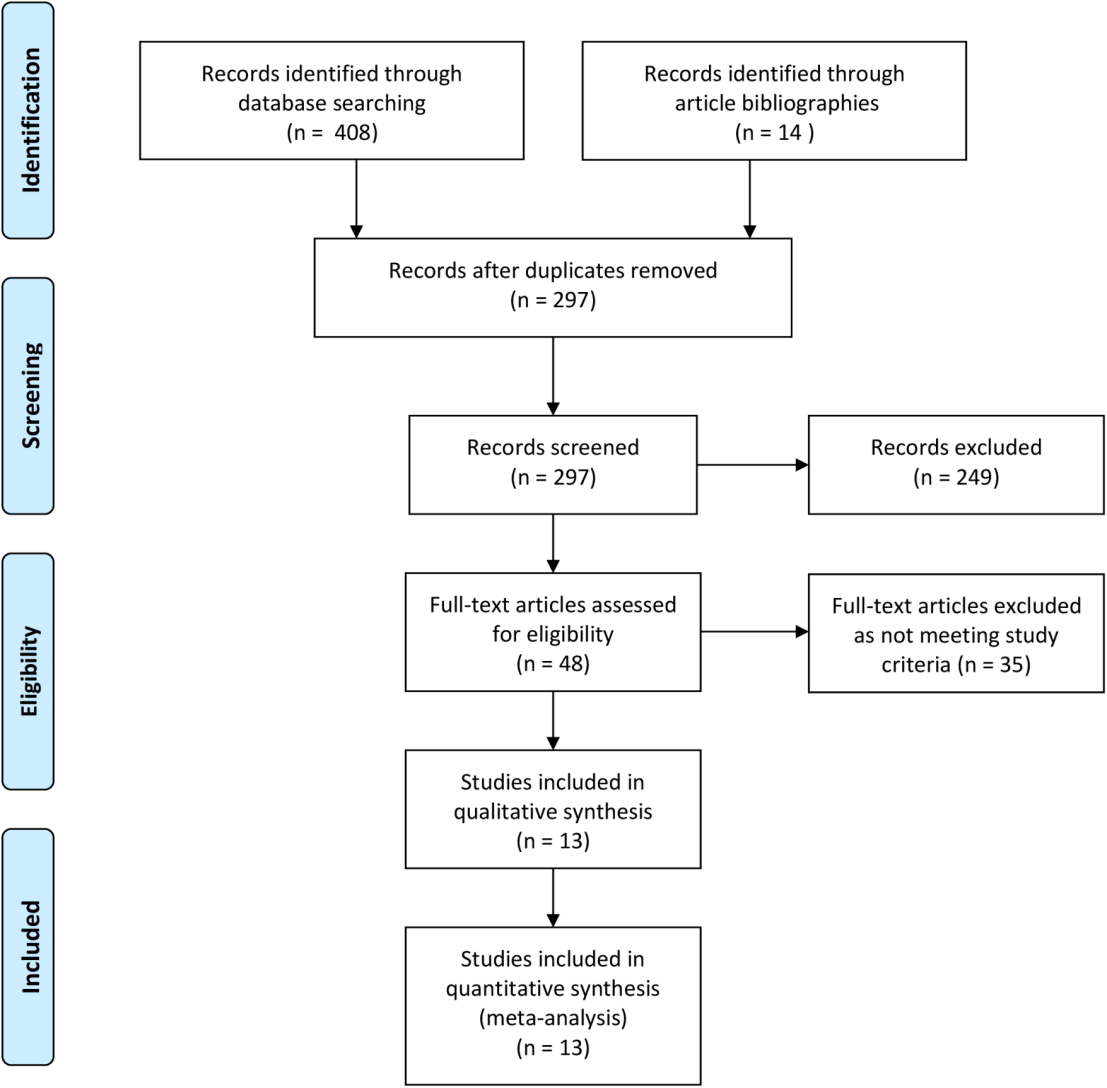
Flow-diagram for screening of articles.
