# Supplementary material for: Smoking, Suicidality and Psychosis: A Systematic Meta-Analysis
Source: PLoS One. 2015 Sep 15;10(9):e0138147. doi: 10.1371/journal.pone.0138147 (PMC4570823; doi:10.1371/journal.pone.0138147)
Supplement: S1 Table — (DOC) [file pone.0138147.s002.doc]

Table 3: Summary of Quality Ratings

| Study | Type of Study | Sample size and Diagnosis | Outcome studied  Suicide Attempt (SA)  Suicide Ideation (SI) | Lifetime  Or  Current  Or  Both |
| --- | --- | --- | --- | --- |
| Altamura et al 2003 | Cohort | 103 patients with schizophrenia or schizoaffective disorder | SA | During the period of observation |
| Altamura et al 2007 | Cross-sectional | 980 patients with DSM IV diagnosis of schizophrenia or schizoaffective disorder | Both SA and SI | Lifetime |
| Andriopoulos et al 2011 | Case-control | 106 patients with schizophrenia admitted to psych department of medical school in Patras | Both SA and SI | The data were segregated into 3 periods: (1) the last month before admission (current episode); (2) the past period, from the first psychotic episode until the current evaluation (lifetime after psychosis onset); and (3) the prodromal period. |
| Baek et al 2013 | Cross-sectional | 1643 patients with diagnosis of Bipolar Disorder I and II | Both SA and SI | during the worst period of time having “low mood” lasted more than 2 weeks |
| Baethge et al 2009 | Cohort | 352 patients with Bipolar disorder | SA | Lifetime |
| Gutierrez-Rojas et al 2012 | Cross-sectional | 108 patients with Bipolar Disorder | SA | Lifetime |
| Iancu et al 2006 | Case-control | 24 schizophrenia patients who never smoked and 37 patients who smoke at least 20 cigarettes a day for at least 5 years | Both SA and SI | Current and Lifetime SI  Lifetime SA |
| Jarbin et al 2004 | Cohort | 88 consecutive patients with DSM IV diagnosis of schizophrenia spectrum disorder | SA | Lifetime |
| Kanwar et al 2013 | Cross-sectional | 920 patients with schizophrenia | SA | Lifetime |
| Kao et al 2011 | Cross-sectional | 95 patients with DSM IV diagnosis of schizophrenia | Both SA and SI | Current SI and Lifetime SA |
| Ostacher et al 2006 | Cohort | 399 patients with bipolar disorder | SA | Lifetime |
| Ostacher et al 2009 | Cohort | 116 patients with bipolar disorder | Both SA and SI | Lifetime and Current |
| Sankaranarayanan et al 2014 | Cross-sectional | 1812 patients with diagnosis of schizophrenia or schizoaffective disorder, BPAD, psychotic depression and other psychosis | Both SA and SI | Lifetime and Current |
