# Supplementary material for: Smoking, Suicidality and Psychosis: A Systematic Meta-Analysis
Source: PLoS One. 2015 Sep 15;10(9):e0138147. doi: 10.1371/journal.pone.0138147 (PMC4570823; doi:10.1371/journal.pone.0138147)
Supplement: S2 Table — (DOC) [file pone.0138147.s003.doc]

Table 3: Guide to Quality Ratings

| Item | Sub-items |  |
| --- | --- | --- |
| Type of Study |  | Cross-sectional=1  Case-control=2  Cohort=3 |
| Aims, Objectives and Hypothesis |  | No clear aims or hypothesis=0  Aims but not hypothesis=1  Aims and hypothesis=2 |
| Internal Validity |  |  |
|  | Blindness of ratings | No=0  Yes=1 |
|  | Clarity on inclusion and exclusion criteria | No=0  Yes=1 |
|  | For cohort studies:  Describes drop-out rates (should be less than 10%)  Describes fixed time periods for assessment | No=-1  Yes=1  No=-1  Yes=1 |
|  | For case-control studies:  Cases and controls from the same population | No=0  Yes=1 |
|  | Accounted for at least 10 confounders | No=0  Yes=1 |
| External Validity | Describes setting | No=0  Yes=1 |
|  | Describes diagnoses studied | No=0  Yes=1 |
|  | Describes who made the diagnosis or assessments | No=0  Yes=1 |
|  | Describes how diagnosis or assessments were made | No=0  Yes=1 |
|  | Defines what suicidal behaviors were studied | No=0  Yes=1 |
|  | Describes how suicidality was measure | No=0  Case-record review=1  Using standardised measures=2 |
| Statistical Validity | Describes rates of suicidal behaviors | No=0  Yes=1 |
|  | Describes sample size calculation or power calculation | No=0  Yes=1 |
|  | At least 10 events per variable | No=0  Yes=1 |
|  | Multivariate analysis used | No=0  Yes=1 |
|  | Describes adjusted Odds Ratio | No=0  Yes=1 |
