# Supplementary material for: Smoking, Suicidality and Psychosis: A Systematic Meta-Analysis
Source: PLoS One. 2015 Sep 15;10(9):e0138147. doi: 10.1371/journal.pone.0138147 (PMC4570823; doi:10.1371/journal.pone.0138147)
Supplement: S3 Table — (DOC) [file pone.0138147.s004.doc]

Table 4: Summary of Quality Ratings

| Study | Type of study  (Maximum=3) | Aims and Hypothesis  (Maximum=2) | Internal Validity  (Maximum=6) | External Validity  (Maximum=7) | Statistical Validity  (Maximum=5) | Final Score  (Maximum=23) | Adjusted Score  (Maximum=23)  ˃16=excellent  11-15=good  6-10=fair  <5=poor | Ranking |
| --- | --- | --- | --- | --- | --- | --- | --- | --- |
| Altamura et al 2003 | 1 | 1 | 1 | 6 | 4 | 13 | 13 | 7 |
| Altamura et al 2007 | 1 | 1 | 0 | 3 | 2 | 7 | 6 | 13 |
| Andriopoulos et al 2011 | 2 | 1 | 2 | 6 | 4 | 15 | 15 | 2 |
| Baek et al 2013 | 1 | 1 | 1 | 6 | 4 | 13 | 13 | 6 |
| Baethge et al 2009 | 3 | 1 | -1 | 6 | 4 | 13 | 12 | 8 |
| Gutierrez-Rojas et al 2012 | 1 | 1 | 2 | 5 | 3 | 12 | 12 | 9 |
| Iancu et al 2006 | 2 | 2 | 2 | 6 | 2 | 14 | 14 | 5 |
| Jarbin et al 2004 | 3 | 1 | 1 | 6 | 3 | 14 | 14 | 4 |
| Kanwar et al 2013 | 1 | 1 | 0 | 4 | 3 | 9 | 8 | 12 |
| Kao et al 2011 | 1 | 2 | 0 | 6 | 2 | 11 | 10 | 11 |
| Ostacher et al 2006 | 3 | 2 | -2 | 6 | 4 | 12 | 11 | 10 |
| Ostacher et al 2009 | 3 | 2 | 2 | 7 | 5 | 19 | 19 | 1 |
| Sankaranarayanan et al 2014 | 1 | 2 | 1 | 6 | 5 | 15 | 15 | 3 |
